# MHC class II transactivator CIITA inhibits Tax-2-mediated HTLV-2 LTR transactivation and viral replication by binding to, and affecting Tax-2 intracellular localization

**DOI:** 10.1186/1742-4690-8-S1-A172

**Published:** 2011-06-06

**Authors:** Chiara Orlandi, Greta Forlani, Giovanna Tosi, Roberto S Accolla

**Affiliations:** 1 Department of Experimental Medicine, University of Insubria, Varese, 21100, Italy

## 

CIITA, the MHC class II transactivator, inhibits the transcriptional function of HTLV-2 (Human T cell Lymphotropic Virus 2) Tax-2 viral transactivator and, consequently, the replication of the virus in human target cells. Here we demonstrate that CIITA and Tax-2 interact in vivo and we identified at least two independent regions, at the 1-252 N-term and at the 410-1130 C-term, respectively, necessary for this interaction, although only the N-term region mediates Tax-2 functional inhibition. Intracellular localization experiments in 293T cells demonstrate that CIITA and Tax-2, when expressed alone, are present both in the cytoplasm and in the nucleus; when co-expressed, however, Tax-2 mostly co-localize with CIITA in the cytoplasm and around the nuclear membrane. The remaining nuclear Tax-2, also co-localize with CIITA. Interestingly, when CIITA nucleus-cytoplasm shuttling is blocked by leptomycin B treatment, most of the Tax-2 molecules are also blocked and co-localize with CIITA in the nucleus, suggesting that direct CIITA-Tax-2 binding does not preclude Tax-2 entry into the nucleus.

Finally, the nuclear factor NF-YB, a CIITA-binding nuclear protein necessary for the MHC class II gene promoter activation, whose overexpression inhibits Tax-2-mediated HTLV-2 LTR transactivation, also strongly binds to Tax-2 in absence of CIITA. Notably, although endogenous NF-YB does not inhibit Tax-2-dependent HTLV-2 LTR transactivation, it still binds to Tax-2, and in presence of CIITA, this binding seems to increase. Taken together these results strongly suggest that that CIITA could inhibit Tax-2 by binding the viral transactivator both directly in the cytoplasm or through a tripartite interaction with NF-YB in the nucleus.

